# Association between serum calcium and prognosis in patients with acute ischemic stroke in ICU: analysis of the MIMIC-IV database

**DOI:** 10.1186/s12871-024-02528-3

**Published:** 2024-04-12

**Authors:** Caijiao Wu, Xiaorong Li, Jiaxing Li, Ruiling Huo, Huihan Zhao, Yanping Ying

**Affiliations:** 1 Department of Dermatology and Venereology, the First Affiliated Hospital of Guangxi Medical University, Nanning, 530021 Guangxi China; 2 Department of Critical Care Medicine, the First Affiliated Hospital of Guangxi Medical University, Nanning, Guangxi China; 3 Department of Neurology Intensive Care Unit, the First Affiliated Hospital of Guangxi Medical University, Nanning, Guangxi China; 4 Department of Nursing, the First Affiliated Hospital of Guangxi Medical University, Nanning, 530021 Guangxi China

**Keywords:** Serum Ca, Mortality, Ischemic stroke, Intensive care unit, MIMIC-IV

## Abstract

**Background:**

While serum Ca has proven to be a reliable predictor of mortality across various diseases, its connection with the clinical outcomes of ischemic stroke (IS) remains inconclusive. Our research aimed to explore the relationships between serum total Ca (tCa) and serum ionized Ca (iCa) and mortality among acute IS (AIS) patients.

**Methods:**

We gathered data from 1773 AIS patients in the Medical Information Mart for Intensive Care Database IV, including baseline demographic data, comorbidities, vital signs, laboratory-based data, and scoring systems. Endpoints for the study encompassed 30-d, 90-d, and 365-d all-cause mortalities. Employing restricted cubic spline Cox regression, we explored potential nonlinear relationships between admission serum iCa and tCa levels and mortality. Participants were categorized into four groups based on serum iCa and tCa quartiles. Multivariable Cox regression analysis was then conducted to evaluate the independent association of iCa and tCa quartiles with all-cause mortality.

**Results:**

The restricted cubic spline revealed a U-shaped association between iCa and 30-d and 90-d mortality (*P*<0.05), while the relationship between iCa and 365-d mortality was linear (*P*<0.05). After adjusting for confounders, multivariable Cox analysis demonstrated that the lowest serum iCa level quartile was independently associated with increased risks of 30-d, 90-d, and 365-d mortality. Similarly, the highest serum iCa level quartile was independently associated with increased risks of 30-d and 90-d mortality, but not 365-d mortality. Notably, serum tCa level showed no association with increased risks of 30-d, 90-d, and 365-d mortality.

**Conclusions:**

Our findings suggest that serum iCa, rather than tCa, is linked to ischemic stroke prognosis. Both high and low serum iCa levels are associated with poor short-term prognosis, while only low serum iCa is associated with poor long-term prognosis in AIS patients.

## Background

Stroke, marked by high disability, morbidity, and mortality rates, presents a significant global health challenge [1]. With 12.2 million incident cases, 101 million prevalent cases, and 6.55 million deaths attributed to stroke, the urgency for effective predictive measures is evident [2]. Amongst stroke types, ischemic stroke (IS) stands as the most common, constituting 70% of all stroke cases [2, 3]. The absolute global deaths due to IS stand at 3.29 million, with predictions hinting at a potential increase to 4.9 million by 2030 [3]. Recognizing the gravity of these statistics, the identification of an early, easily accessible predictor becomes crucial for informed clinical decisions and the implementation of appropriate treatments.

Calcium (Ca), the most abundant mineral with the human body, has been shown to modulate numerous physiological processes, including nerve transmission, cell membrane stability, coagulation, muscle contraction, fluid balance regulation, endocrine, and immune functions [4, 5]. In serum, Ca exists in three fractions: ionized, bound to plasma proteins, and chelated to serum anions [6]. The body meticulously controls serum Ca levels within a narrow range under normal physiological conditions. Dyscalcemia has been associated with the risks of cerebrovascular and cardiovascular diseases [7, 8].

Despite the paramount importance of Ca, studies investigating the link between serum Ca levels and outcomes in Acute Ischemic Stroke (AIS) are scarce and yield conflicting results [9–18]. Some report correlations between both low and high serum Ca levels and poor IS outcomes [11–13], while others note a non-linear association between serum total Ca (tCa) levels and all-cause death over a year [15]. Notably, the study by Ramya et al. [18] highlights an inverse association between serum ionized Ca (iCa) and AIS prognosis. Adding to the complexity, certain studies have reported no significant relationships between serum Ca levels and AIS outcomes [10, 16, 17]. The variations in Ca markers and study endpoints across these studies raise the question of whether the contradictory findings stem from these differences.

Therefore, our study was designed to unravel the potential association between admission serum iCa and tCa levels and the risks of all-cause mortality in AIS patients. By utilizing extensive real-world databases, we aim to comprehensively explore both long-term and short-term outcomes, providing a holistic understanding of the intricate relationships between serum Ca and AIS outcomes.

## Materials and methods

### Data source

This retrospective investigation utilized the Medical Information Mart for Intensive Care (MIMIC)-IV database (v2.0), an iteration succeeding MIMIC-III. The database, aligned with the Health Insurance Portability and Accountability Act Safe Harbor provision, ensures deidentification. MIMIC-IV encompasses robust clinical data from 70,000 adult intensive care unit(ICU) patients at BIDMC between 2008 and 2019. Approval for employing the MIMIC-IV databases was granted by the Institutional Review Board of the Beth Israel Deaconess Medical Center and Massachusetts Institute of Technology. All patient data within the database is anonymized, obviating the need for informed consent. In adherence to the ethical standards articulated in the 1964 Declaration of Helsinki and its subsequent amendments, the study was conducted. Access to the database was secured following the completion of the National Institutes of Health Web-based training course and the Protecting Human Research Participants examination (No. 52784856).

### Study population

**Fig. 1:**
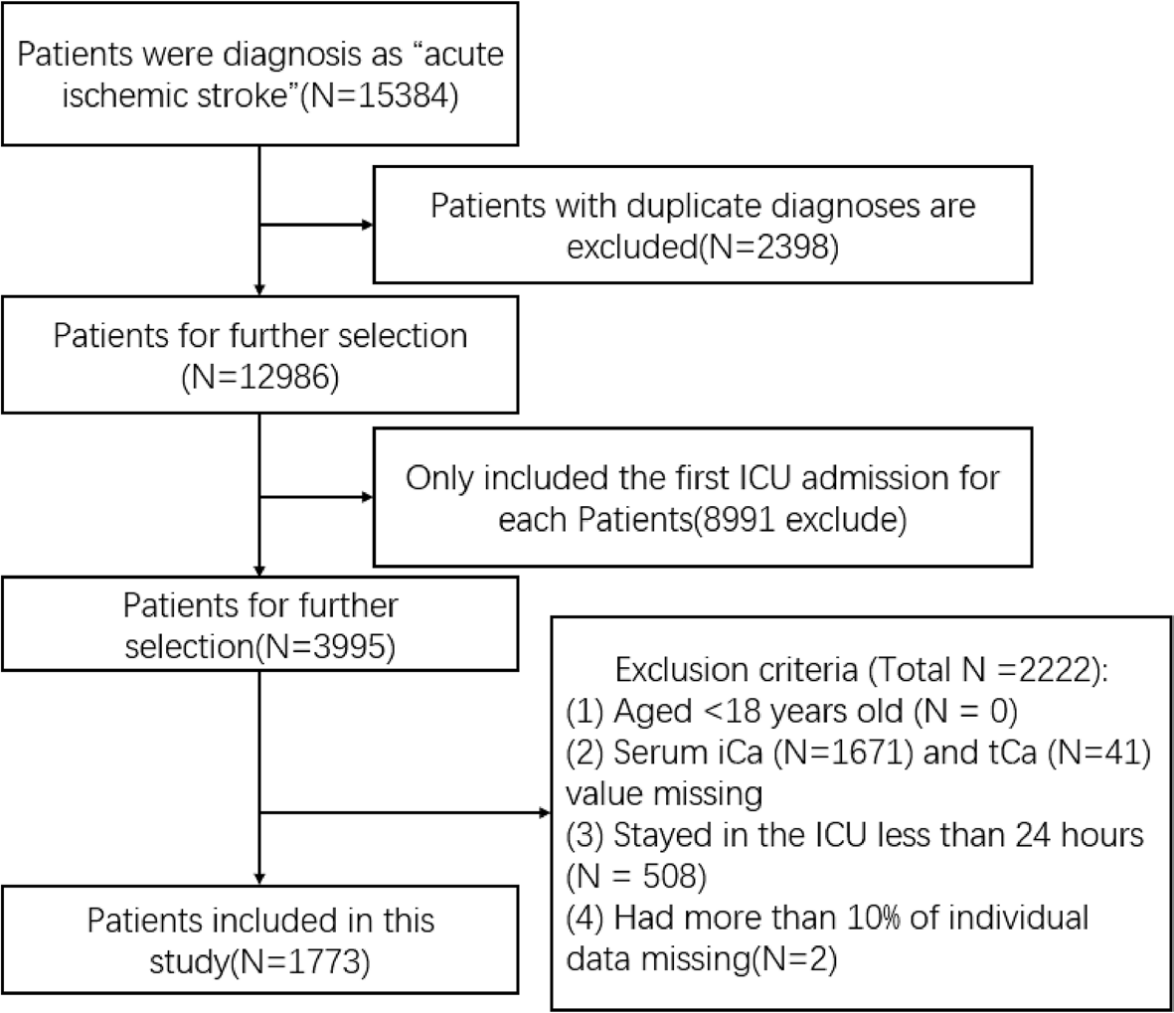
Flowchart of study population

Between 2008 and 2019, we identified individuals in the MIMIC-IV database meeting the following criteria: adults (aged ≥ 18 years) diagnosed with ischemic stroke, as indicated by ICD-9 codes 433/434/436/437.0/437.1, or ICD-10 codes I63/I65/I66 (Fig. 1). Only the initial ICU admission date was considered for patients with multiple ICU admissions. Exclusions were made for individuals with (i) incomplete serum iCa and tCa data; (ii) ICU stays of less than 24 hours; (iii) more than 10% missing individual data.

### Data extraction, preparation, and definitions

SQL (Structured Query Language) programming in Navicat Premium 15.0 software was used for data extraction. Patient characteristics were extracted as follows: (1) baseline demographic variables: age, sex, race. (2) vital signs (initial values on the 1^st^ day of the ICU): mean artery pressure (MAP), heart rate, percutaneous oxygen saturation (SpO2), temperature, and respiratory rate. (3) laboratory data (from the first record after ICU admission): tCa, iCa, potassium, phosphate, chloride, sodium, lactate, bicarbonate, creatinine, pH, white blood cell count (WBC), platelet, hemoglobin, magnesium, serum-glucose, BUN, and estimated glomerular filtration rate (eGFR; measured through the CKD-Epi formula). (4) comorbidities (processed into categorical variables for statistical analysis): hypertension, hyperlipidemia, coronary artery disease (CAD), diabetes, chronic pulmonary disease, congestive heart failure, liver disease, chronic kidney disease (CKD), malignancy, atrial fibrillation, rheumatic disease, heart failure, renal failure. (5) Severity scoring system (measured from the first record after the ICU admission: the Sequential Organ Failure Assessment (SOFA), acute physiology score III (APS III), the Glasgow Coma Scale (GCS), and systemic inflammatory response syndrome (SIRS). Additionally, treatment information data were acquired, including renal replacement treatment (RRT), mechanical ventilation, mechanical thrombectomy and thrombolytic drugs.

### Determining the threshold values of serum tCa and iCa levels

Serum Ca levels were assigned to 4 groups based on the quartiles (Q1-Q4) of their concentrations.

### Study outcomes

The primary outcome of this research was 30-d all-cause mortality, whereas the secondary outcomes encompassed 90-d and 365-d all-cause mortality.

### Statistical analysis

Continuous data were presented as mean±standard deviation or median (interquartile range), while categorical data were expressed as numbers (percentages). To assess data normality, the Shapiro–Wilk test was employed. One-way ANOVA and Kruskal–Wallis H tests were conducted for continuous data with normal and skewed distribution, respectively, while Pearson’s Chi-square (χ2) test or Fisher’s exact test was utilized for categorical data.

Potential nonlinear associations between serum Ca levels and 30-d, 90-d, and 365-d mortality were examined using restricted cubic splines. Analyses were adjusted for multiple variables, with trimming of the highest and lowest 0.5% for serum Ca levels. Knots were positioned at the 5/25/75/95th percentiles for serum Ca measures. Likelihood ratio tests were conducted to test for nonlinearity.

The patients were assigned to 4 groups based on serum Ca levels (iCa<1.07 mmol/L, 1.07 mmol/L*≤*iCa<1.12 mmol/L, 1.12 mmol/L≤iCa<1.17 mmol/L, and iCa*≥*1.17 mmol/L, tCa<7.9 mg/dl, 7.9 mg/dL*≤*tCa<8.4 mg/dl, 8.4 mg/dL*≤*tCa<9.0 mg/dl, and tCa*≥*9.0 mg/dl). Log-rank tests and Kaplan–Meier methods estimated the absolute risk of events for each group. Univariate and multivariate Cox analyses identified associations between serum Ca quartiles and 30-d, 90-d, and 365-d mortality.

In the Cox regression models, Model I adjusted for gender, race, and age; Model II further adjusted for MAP, respiratory rate, heart rate, temperature, SpO2, SIRS, APS III, liver disease, malignancy, renal failure, hyperlipidemia, mechanical ventilation, RRT, mechanical thrombectomy, and thrombolytic drugs; Model III further adjusted for serum glucose, hemoglobin, platelet, WBC, creatinine, BUN, sodium, chloride, magnesium, bicarbonate, lactate, pH, phosphate, and eGFR based on Model II.

The Q3 of 1.12≤iCa<1.17 mmol/L and 8.4≤tCa<9.0 mg/dL served as the reference group. HRs, accompanied by 95% CIs were disclosed in multivariable Cox regression models. Adjustment for potential confounders was made, with selection based on P-values ≤ 0.05 from univariable analysis. Missing data in the MIMIC database were addressed using multiple imputation, according to 5 replications and a chained equation approach in the R MI procedure. A two-tailed *P*<0.05 was deemed statistically significant, and all tests were conducted using R software (v4.2.1).

## Results

### Baseline characteristics

In total, 1773 IS patients admitted to the ICU were identified in the MIMIC-IV database based on the selection criteria (Fig. 1). Table 1 presents the demographic features of the subjects, categorized according to serum iCa quartiles. The median age of the participants was 71.4 (61.7-80.2) years, with 1020 (57.5%) subjects being male. The median admission serum tCa and iCa levels were 1.12 (1.07-1.17) mmol/L and 8.40 (7.90-9.00) mg/dL, respectively. Next, serum iCa levels were assigned to the Q1 group (iCa<1.07 mmol/L), Q2 group (1.07≤iCa<1.12 mmol/L), Q3 group (1.12≤ iCa<1.17 mmol/L), and Q4 group (1.17 mmol/L≤iCa). Similarly, serum tCa levels were categorized as follows: Q1 group (tCa<7.9 mg/dL), Q2 group (7.9≤tCa<8.4 mg/dL), Q3 group (8.4≤tCa<9.0 mg/dL), and Q4 group (9.0 mg/dL≤tCa). Within these groups, 428 patients were in Q1 group (iCa<1.07 mmol/L), 401 patients in Q2 group (1.07≤iCa<1.12 mmol/L), 436 patients in Q3 group (1.11≤iCa<1.17 mmol/L), and 508 patients in Q4 group (1.17 mmol/L≤iCa). Compared with those in Q2-4 groups, patients in Q1 group were more likely to exhibit higher MAP, respiratory rate, heart rate, temperature, serum-glucose, hemoglobin, platelet, WBC, creatinine, BUN, and phosphate. Additionally, they had a higher prevalence of comorbidities, including liver disease and CKD. Furthermore, this group was more inclined to receive interventions such as to receive mechanical ventilation, RRT, and thrombolytic drugs.

**Table 1:** Baseline characteristics of the patients according to quartiles of serum iCa levels

iCa levels (mmol/L)						
**Characteristics**	Overall (*N*=1773)	Q1(iCa<1.07) (*N*=428)	Q2(1.07≤iCa<1.12) (*N*=401)	Q3(1.12≤iCa<1.17) (*N*=436)	Q4(1.17≤iCa) (*N*=508)	*P* value
Age (years)	71.4(61.7-80.2)	68.8(57.3-77.8)	72.1(61.5-81.4)	72.1(62.6-81.7)	72.4(64.1-80.6)	<0.001
**Gender, n**						0.519
Female	753(42.5%)	193(45.1%)	172(42.9%)	175(40.1%)	213(41.9%)	
Male	1020(57.5%)	235(54.9%)	229(57.1%)	261(59.9%)	295(58.1%)	
**Race,n**						0.067
White	1131(63.8%)	251(58.6%)	261(65.1%)	295(67.7%)	324(63.8%)	
Black	133(7.5%)	35(8.2%)	22(5.5%)	27(6.2%)	49(9.6%)	
Asian	38(2.1%)	13(3.0%)	10(2.5%)	6(1.4%)	9(1.8%)	
Other	471(26.6%)	129(30.1%)	108(26.9%)	108(24.8%)	126(24.8%)	
**Vital signs**						
MAP (mmHg)	85.7(74.0-99.0)	88.3(75.7-99.8)	87.3(75.7-100.0)	83.5(72.0-97.1)	83.3(73.0-99.3)	0.003
Respiratory rate(breath/min)	17.0(15.0-22.0)	19.0(16.0-24.0)	17.0(15.0-22.0)	16.0(14.0-21.0)	16.0(14.0-20.0)	<0.001
Heart rate(beats/min)	82.0(72.0-95.0)	86.0(73.0-100.0)	82.0(72.0-97.0)	80.0(70.0-93.0)	80.0(73.0-92.0)	0.014
Temperature (°C)	36.7(36.3-37.1)	36.7(36.4-37.1)	36.7(36.3-37.1)	36.6(36.3-37.1)	36.6(36.2-37.0)	<0.001
SpO2 (%)	99.0(97.0-100.0)	99.0(96.0-100.0)	99.0(96.0-100.0)	100.0(97.0-100.0)	100.0(97.0-100)	<0.001
**Laboratory-based data**						
Serum-glucose(mg/dL)	131.0(109.0-167.0)	143.0(114.0-182.0)	131.0(110.0-162.0)	129.0(107.0-159.0)	127.0(106.0-161.0)	<0.001
Hemoglobin (g/dL)	10.6(8.9-12.3)	10.9(9.10-12.4)	10.6(9.10-12.3)	10.4(8.68-12.2)	10.4(8.70-12.1)	0.025
Platelet (10*9/L)	182.0(132.0-245.0)	196.0(137.0-263.0)	182.0(131.0-251.0)	175.0(133.0-238.0)	173.0(129.0-232.0)	0.025
WBC (10*9/L)	11.8(8.70-15.6)	12.5(9.0-16.5)	12.0(8.8-15.8)	11.1(8.10-14.8)	11.8(8.58-15.1)	0.006
tCa(mg/dL)	8.4 (7.9-9.0)	8.0(7.5-8.6)	8.3(7.9-8.8)	8.5 (8.1-9.0)	8.7 (8.3-9.3)	<0.001
Creatinine(mg/dL)	1.0 (0.7-1.3)	1.0(0.7-1.7)	0.9 (0.7-1.3)	0.9 (0.7-1.2)	0.9 (0.7-1.2)	0.004
eGFR(mL/min/1.73 m )	79.6(51.5-111.0)	75.7(46.8-113.0)	79.6(50.7-109.0)	79.1(52.7-109.0)	81.5(54.1-114.0)	0.234
BUN (mg/dL)	19.0(13.0-28.0)	20.5(13.0-34.0)	18.0(13.0-27.0)	18.0(14.0-26.0)	19.0(13.8-27.0)	0.010
Sodium (mmol/L)	139.0(136.0-142.0)	139.0(136.0-141.0)	139.0(136.0-141.0)	139.0(136.0-142.0)	140.0(137.0-142.0)	<0.001
Potassium (mmol/L)	4.1 (3.8-4.6)	4.1(3.7-4.5)	4.1 (3.7-4.5)	4.2 (3.8-4.6)	4.2 (3.8-4.6)	0.001
Chloride (mmol/L)	106.0(102.0-110.0)	105.0(101.0-109.0)	106.0(101.0-109.0)	106.0(102.0-110.0)	108.0(104.0-111.0)	<0.001
Magnesium(mg/dL)	2.0 (1.8-2.3)	1.9(1.7-2.2)	2.0 (1.7-2.2)	2.0 (1.8-2.3)	2.1 (1.8-2.5)	<0.001
Bicarbonate (mmol/L)	23.0(20.0-25.0)	22.0(19.0-24.0)	22.0(20.0-24.0)	23.0(21.0-25.0)	23.0(21.0-25.0)	<0.001
Lactate (mmol/L)	1.7 (1.2-2.5)	1.7(1.3-2.8)	1.7 (1.2-2.5)	1.6 (1.1-2.3)	1.8 (1.3-2.6)	0.003
pH level	7.4 (7.3-7.4)	7.4(7.3-7.4)	7.4 (7.3-7.4)	7.4 (7.4-7.5)	7.4(7.3-7.4)	0.001
Phosphate(mg/dL)	3.5 (2.9-4.2)	3.6 (2.9-4.6)	3.5 (2.9-4.1)	3.5 (2.9-4.1)	3.4 (2.8-4.1)	0.002
**Comorbidities,n(%)**						
CAD	488(27.5%)	116(27.1%)	106(26.4%)	130(29.8%)	136(26.8%)	0.666
Congestive heart failure	546(30.8%)	136(31.8%)	129(32.2%)	139(31.9%)	142(28.0%)	0.438
Chronic pulmonary disease	437(24.6%)	93(21.7%)	109(27.2%)	116(26.6%)	119(23.4%)	0.199
Rheumatic disease	46(2.6%)	12(2.8%)	11(2.7%)	6(1.4%)	17(3.3%)	0.283
Liver disease	120(6.8%)	42(9.8%)	25(6.2%)	29(6.7%)	24(4.7%)	0.020
Diabetes	636(35.9%)	142(33.2%)	126(31.4%)	161(36.9%)	207(40.7%)	0.016
Malignancy	156(8.8%)	40(9.3%)	37(9.2%)	44(10.1%)	35(6.9%)	0.325
Hypertension	959(54.1%)	208(48.6%)	222(55.4%)	242(55.5%)	287(56.5%)	0.072
Heart failure	517(29.2%)	133(31.1%)	122(30.4%)	131(30.0%)	131(25.8%)	0.258
Atrial fibrillation	709(40.0%)	149(34.8%)	172(42.9%)	174(39.9%)	214(42.1%)	0.067
Renal failure	204(11.5%)	42(9.8%)	55(13.7%)	47(10.8%)	60(11.8%)	0.334
CKD	75(4.2%)	35(8.2%)	10(2.5%)	14(3.2%)	16 3.1%)	<0.001
Hyperlipidemia	870(49.1%)	163(38.1%)	199(49.6%)	227(52.1%)	281(55.3%)	<0.001
**Scoring system**						
GCS	15.0(15.0-15.0)	15.0(15.0-15.0)	15.0(15.0-15.0)	15.0(15.0-15.0)	15.0(15.0-15.0)	0.297
APS III	50.0(35.0-71.0)	58.0(40.0-82.0)	50.0(35.0-68.0)	47.0(35.0-66.0)	46.0(33.0-70.0)	<0.001
SOFA	2.0 (1.0-4.0)	2.0 (1.0-4.0)	2.0 (1. 0-4.0)	2.00(1.0-4.0)	2.0 (1. 0-4.0)	0.351
SIRS	3.0 (2.0-3.0)	3.0 (2.0-3.0)	3.0 (2.0-3.0)	3.00(2.0-3.0)	3.0 (2. 0-3.0)	0.004
**Treatment information,n(%)**						
Mechanical ventilation	262(14.8%)	80(18.7%)	67(16.7%)	53(12.2%)	62(12.2%)	0.010
RRT	123(6.9%)	62(14.5%)	18(4.5%)	21(4.8%)	22(4.3%)	<0.001
Mechanical thrombectomy	47(2.7%)	14(3.3%)	12(3.0%)	15(3.4%)	6(1.2%)	0.106
Thrombolytic drugs	288(16.2%)	92 21.5%)	64(16.0%)	64(14.7%)	68(13.4%)	0.006
Hospital length of stay,day	11.9(7.24-19.9)	13.7(7.97-22.5)	12.2(7.92-19.7)	11.9(7.16-20.6)	10.7(6.6-16.9)	<0.001
ICU length of stay,day	4.35(2.21-9.09)	5.84(2.79-11.5)	4.97(2.31-9.32)	4.1(2.1-8.2)	3.86(2.1-7.9)	<0.001

Values are expressed as the median (IQR) or n (%)

*Abbreviations*: *iCa* ionized calcium, *MAP* mean artery pressure, *SpO2* saturation of peripheral oxygen, *WBC* White Blood Cell, *tCa* total calcium, *eGFR* Estimated glomerular filtration rate, *BUN* Blood Urea Nitrogen, *PH* Potential of hydrogen, *CAD* Coronary artery disease, *CKD* Chronic kidney disease, *GCS* Glasgow Coma Scale, *APS III* Acute physiology score III, *SOFA* Sequential Organ Failure Assessment, *SIRS* Systemic inflammatory response syndrome, *RRT* Renal replacement treatment, *IQR* interquartile range

### Relationships between serum Ca levels and mortality

**Fig. 2:**
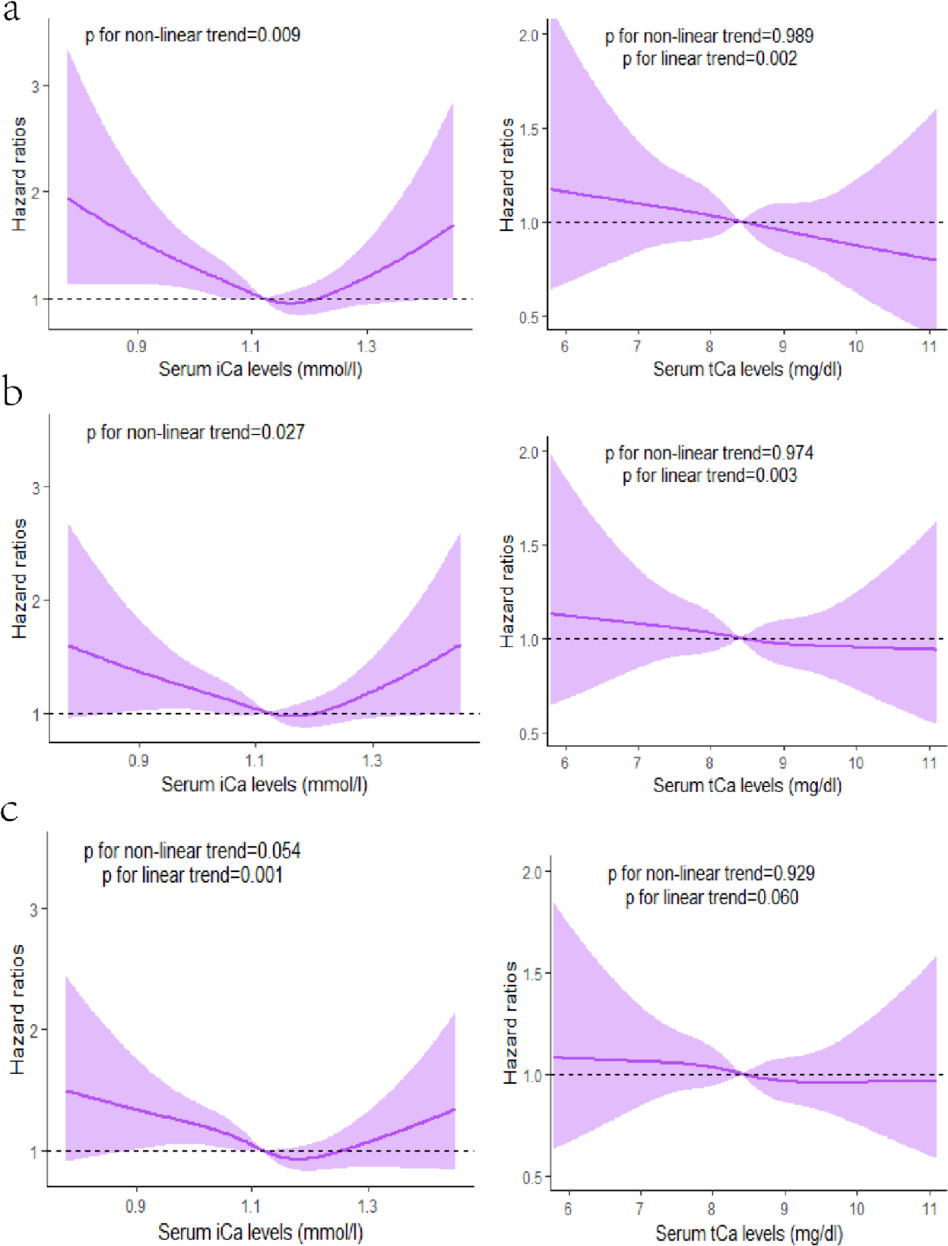
Association of admission serum calcium levels with mortality in restricted cubic spline models. **a** Serum iCa levels and 30-day mortality (left panel) Serum tCa levels and 30-day mortality (right panel). **b** Serum iCa levels and 90-day mortality (left panel) Serum tCa levels and 90-day mortality (right panel). **c** Serum iCa levels and 365-day mortality (left panel) Serum tCa levels and 365-day mortality (right panel). The purple lines and Shaded areas represent the estimated HR and the 95% CI, respectively.Abbreviation: HR: hazard ratio; CI: confidence interval; iCa: ionized calcium; tCa: total calcium

In Fig. 2, the results of multivariable-adjusted restricted cubic spline analyses revealed U-shaped associations between serum Ca levels (iCa) with 30-d and 90-d mortality. Nonlinear trends were observed for iCa with both 30-d and 90-d mortality (*P*<0.05). Notably, the lowest risk of mortality was identified at 1.16 mmol/L for iCa. Specifically, when iCa was less than 1.16 mmol/L, the risk of mortality reduced with increasing iCa concentration. Conversely, when iCa exceeded 1.16 mmol/L, the risk of mortality increased with iCa concentration. However, the relationship between iCa and 365-d mortality demonstrated linearity (*P*<0.05). Furthermore, the analyses highlighted significant linear relationships between tCa and 30-d, 90-d, and 365-d mortality (*P*>0.05).

### Survival analysis

Among the 1773 IS patients analyzed, 23.0% (407/1773) died during the first 30 days, 29.7% (527/1773) died during the first 90 days, and 36.5% (647/1773) succumbed over the 1-year follow-up period. Notably, the 30-d mortality rates were 29.2% for serum iCa <1.07 mmol/L, 26.7% for 1.07-1.12 mmol/L, 15.6% for 1.12-1.17 mmol/L, and 21.1% for ≥1.17 mmol/L. The 90-d mortality rates were 37.6% for serum iCa of <1.07 mmol/L, 32.2% for 1.07-1.12 mmol/L, 22.5% for 1.12-1.17 mmol/L, and 27.4% for ≥1.17 mmol/L. The 365-d mortality rates were 44.9% for serum iCa <1.07 mmol/L, 39.9% for 1.07-1.12 mmol/L, 30.3% for 1.12-1.17 mmol/L, and 32.1% for ≥1.17 mmol/L.

**Fig. 3:**
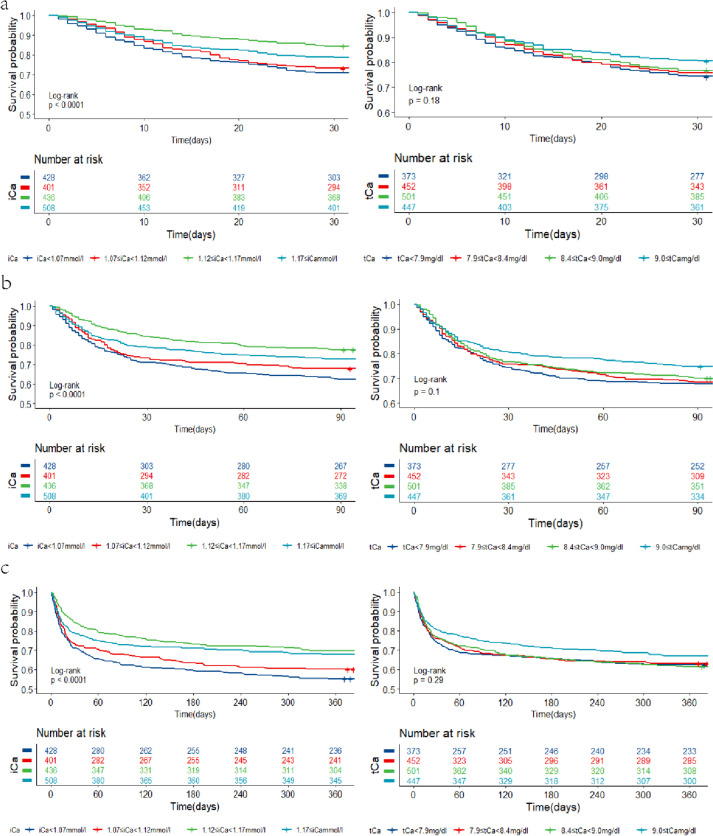
Kaplan–Meier curves of serum calcium level quartiles. **a** Serum iCa and 30-day mortality(left panel), serum tCa and 30-day mortality(right panel). **b** Serum iCa and 90-day mortality(left panel), serum tCa and 90-day mortality(right panel). **c** Serum iCa and 365-day mortality(left panel), serum tCa and 365-day mortality(right panel). iCa: ionized calcium; tCa: total calcium. (left panel) The deep blue line represents iCa < 1.07 mmol/L; the red line represents 1.07 ≤ iCa <1.12 mmol/L; the green line represents 1.12 ≤ iCa < 1.17 mmol/L; the light blue line represents 1.17 mmol/L≤iCa. (right panel) The deep blue line represents tCa < 7.9 mg/dL; the red line represents 7.8 ≤ tCa < 8.4 mg/dL; the green line represents 8.4 ≤ tCa < 9.0 mg/dL; the light blue line represents 9.0 mg/dL≤ tCa

Figure 3 illustrates Kaplan–Meier curves depicting all-cause mortality across serum Ca quartiles. The curves for serum iCa quartiles exhibited significant differences (log-rank test: *P*<0.01 for 30-d, 90-d, and 365-d mortalities), with patients in the lowest serum iCa quartile displaying the highest cumulative incidence of mortality. In contrast, there was no obvious difference in the curves for serum tCa quartiles (log-rank test: *P*>0.05 for 30-d, 90-d, and 365-d mortalities).

### Relationships between serum Ca and clinical outcomes

Serum Ca was selected as the independent variable, while 30-d, 90-d and 365-d mortality as the dependent variables in the multiple regression analysis. Other variables served as covariates to enhance the model's stability, leading to the construction of 4 models (Table 2). In the non-adjusted models, the results indicated that the low serum iCa level quartile (Q1 or Q2) emerged as a significant predictor of 30-d, 90-d, and 365-d mortalities compared to the reference group (Q3). This observation persisted in Model I, even after adjusting for race, gender, and age. Model II, which further adjusted for covariates such as gender, age, race, MAP, respiration rate, heart rate, temperature, SpO2, SIRS, APSIII, liver disease, malignancy, renal failure, hyperlipidemia, mechanical ventilation, RRT, mechanical thrombectomy, and thrombolytic drugs, yielded similar results. The robustness of these findings continued in Model III, which further adjusted for serum-glucose, hemoglobin, platelet, WBC, creatinine, BUN, sodium, chloride, magnesium, bicarbonate, lactate, pH, phosphate, and eGFR on the basis of Model II. Specifically, the outcomes indicated that the high serum iCa level quartile (Q4) significantly predicted 30-d and 90-d mortalities, but not 365-d mortality, compared to the reference group (Q3), after adjusting for potential confounders in Model III. However, both low serum tCa level (Q1 or Q2 vs. Q3) and high serum tCa level (Q4 vs. Q3) were not associated with the incidence of 30-d, 90-d, and 365-d mortality in the four models (*P*>0.05).

**Table 2:** Association between serum iCa and tCa levels and mortality in patients with AIS

Clinical outcomes	Non-adjusted Model HR(95%CI) *P* value		Model I HR(95%CI) *P* value		Model II HR(95%CI) *P* value		Model III HR(95%CI) *P* value	
30-day mortality								
iCa (mmol/L)								
Q1 (iCa<1.07)	2.06(1.54,2.77)	<0.001	2.14(1.59,2.88)	<0.001	1.65(1.22,2.25)	<0.001	1.58(1.16,2.15)	0.004
Q2 (1.07≤iCa< 1.12)	1.83(1.35,2.48)	<0.001	1.84(1.36,2.49)	<0.001	1.64(1.21,2.23)	0.001	1.55(1.13,2.11)	0.006
Q3 (1.12≤iCa< 1.17)	1		1		1		1	
Q4 (1.17≤iCa)	1.43(1.06,1.94)	0.020	1.42(1.05,1.93)	0.023	1.55(1.14,2.12)	0.005	1.53(1.12,2.09)	0.008
tCa (mg/dL)								
Q1 (tCa<7.9)	1.14(0.87,1.50)	0.328	1.21(0.93,1.59)	0.160	0.94(0.71,1.24)	0.64	0.85(0.63,1.14)	0.272
Q2 (7.9≤tCa<8.4)	1.06(0.81,1.38)	0.669	1.09(0.84,1.42)	0.518	1.13(0.87,1.48)	0.36	1.04(0.80,1.37)	0.751
Q3 (8.4≤tCa<9.0)	1		1		1		1	
Q4 (9.0≤tCa)	0.84(0.63,1.10)	0.206	0.81(0.61,1.07)	0.132	0.86(0.65,1.15)	0.31	0.83(0.62,1.10)	0.195
90-day mortality								
iCa (mmol/L)								
Q1 (iCa<1.07)	1.88(1.47,2.42)	<0.001	2.01(1.56,2.59)	<0.001	1.58(1.22,2.04)	<0.001	1.46(1.12,1.90)	0.004
Q2 (1.07≤iCa< 1.12)	1.55(1.20,2.02)	0.001	1.58(1.21,2.05)	<0.001	1.44(1.11,1.88)	0.007	1.36(1.04,1.77)	0.026
Q3 (1.12≤iCa<1.17)	1		1		1		1	
Q4 (1.17≤iCa)	1.28(0.99,1.66)	0.059	1.28(0.99,1.66)	0.063	1.42(1.09,1.84)	<0.01	1.43(1.10,1.87)	0.008
tCa (mg/dL)								
Q1 (tCa<7.9)	1.12(0.88,1.42)	0.355	1.20(0.94,1.53)	0.137	0.91(0.71,1.17)	0.473	0.83(0.64,1.08)	0.175
Q2 (7.9≤tCa<8.4)	1.07(0.85,1.35)	0.546	1.10(0.87,1.38)	0.424	1.16(0.92,1.47)	0.209	1.09(0.86,1.39)	0.455
Q3 (8.4≤tCa<9.0)	1		1		1		1	
Q4 (9.0≤tCa)	0.83(0.65,1.06)	0.133	0.80(0.62,1.02)	0.071	0.87(0.68,1.11)	0.260	0.84(0.65,1.07)	0.16
365-day mortality								
iCa (mmol/L)								
Q1 (iCa<1.07)	1.70(1.36,2.12)	<0.001	1.85(1.48,2.31)	<0.001	1.48(1.18,1.86)	<0.001	1.37(1.09,1.73)	0.008
Q2 (1.07≤iCa<1.12)	1.45(1.15,1.82)	0.002	1.48(1.17,1.86)	<0.001	1.36(1.08,1.72)	0.009	1.30(1.02,1.63)	0.033
Q3 (1.12≤iCa<1.17)	1		1		1		1	
Q4 (1.17≤iCa)	1.11(0.89,1.40)	0.356	1.10(0.88,1.39)	0.403	1.23(0.97,1.55)	0.089	1.22(0.97,1.55)	0.093
tCa (mg/dL)								
Q1 (tCa<7.9)	1.00(0.81,1.25)	0.988	1.08(0.87,1.35)	0.478	0.84(0.67,1.05)	0.127	0.81(0.64,1.03)	0.091
Q2 (7.9≤tCa<8.4)	0.97(0.79,1.20)	0.783	0.98(0.80,1.21)	0.884	1.05(0.85,1.29)	0.663	1.03(0.83,1.28)	0.806
Q3 (8.4≤tCa<9.0)	1		1		1		1	
Q4 (9.0≤tCa)	0.83(0.67,1.03)	0.087	0.79(0.63,0.98)	0.03	0.84(0.68,1.05)	0.128	0.80(0.65,1.00)	0.068

Non-adjusted Model: not adjusted

Mode I: adjusted for gender, race, age

Mode II: adjusted for gender, race, age, MAP, respiratory rate, heart rate, temperature, spo2, SIRS, APS III, liver disease, malignancy, renal failure, hyperlipidemia, mechanical ventilation, RRT, thrombolytic drugs and Mechanical thrombectomy

Model III: adjusted for gender, race, age, MAP, respiratory rate, heart rate, temperature, spo2, SIRS, APS III, liver disease, malignancy, renal failure, hyperlipidemia, mechanical ventilation, RRT, thrombolytic drugs, Mechanical thrombectomy, serum glucose, hemoglobin, platelet, WBC, creatinine, BUN, sodium, chloride, magnesium, bicarbonate, lactate, PH, phosphate and eGFR

## Discussion

Stroke stands as the 2^nd^ leading cause of mortality globally and remains a primary contributor to adult physical disability [2]. Within the spectrum of strokes, IS represents a substantial majority, accounting for 70% of all cases and exhibiting an increased risk of mortality [1, 3]. IS unfolds as a consequence of compromised blood supply to brain tissue, resulting in a reduction of oxygen and glucose levels, ultimately leading to an inadequate production of adenosine triphosphate (ATP) [19]. This energy deficit triggers a cascade of biochemical and physiological events [20], including disturbance of ion homeostasis, neuronal excitotoxicity, peri-infarct depolarization, nitrative and oxidative stress, apoptosis and inflammation [21, 22]. These pathophysiological processes inflict severe damage upon neurons, glia, and endothelial cells, leading to irreversible neuronal injury [1, 23]. The severity of IS correlates with factors such as the size of cerebral infarction, cerebral edema, and hemorrhagic transformation, all of which are linked to unfavorable outcomes in AIS [24–26].

Ca plays a crucial role as a structural component of bone and is involved in various essential functions within the body [27]. Normally, blood Ca level are tightly regulated, maintaining a normal range of tCa concentration (8.6-10.3 mg/dL) or (2.2-2.6 mmol/L) and iCa concentration (4.3-5.1 mg/dL) or (1.1-1.3 mmol/L). Ca ions (Ca2+) homeostasis is imperative for the survival and proper function of neuronal cells [28]. When the intracellular Ca2+ concentration reaches a critical level, it can lead to neuronal damage and cell death [29]. Under normal conditions, Ca ions are primarily reside in the extracellular fluid, with intracellular Ca levels remaining significantly low. However, during an IS event, the deprivation of glucose and oxygen supply to brain tissues results in the immediate failure of ATP-dependent ion channels and pumps, leading to the liberation of potentially harmful levels of excitatory neurotransmitters, followed by the influx of Ca ions [30]. This massive influx of Ca triggers the activation of lethal second messengers and enzymes, mitochondrial dysfunction, inflammatory cell infiltration, and increased free radical generation [31]. These events collectively contribute to neuronal cell death and brain damage, fostering a detrimental feedback loop of further Ca ion influx [31].

To date, there has been limited research exploring the association between serum Ca levels and clinical outcomes in ischemic stroke, with existing findings exhibiting conflicting results [9–18]. Previous research has demonstrated a significant decrease in serum Ca levels in patients who succumbed during hospitalization compared to survivors [32]. Several reports have indicated an inverse relationship between serum Ca levels and both infarct volume and clinical severity in IS cases [14, 18]. Additionally, higher total serum calcium values detected upon admission in acute ischemic stroke patients have been linked to poorer short-term outcomes and high risks of long-term mortality following the acute event [11–13]. Intriguingly, a post-hoc analysis of a prospective longitudinal cohort study, encompassing 784 consecutive AIS patients, unveiled a U-shaped association between serum Ca levels and in-hospital all-cause mortality [15]. On the contrary, some reports have demonstrated almost no obvious association between serum Ca levels and AIS outcomes [10, 16, 17]. Furthermore, a study reviewing data from a double-blind, placebo-controlled, multi-center trial revealed that elevated serum Ca levels at 72 to 96 hours post-stroke predicted greater independence three months after IS, while admission serum Ca levels did not seem to exhibit prognostic significance [33]. These conflicting outcomes may be attributed to variations in IS subtype, serum Ca type, serum Ca measurement methods, and whether Ca correction was applied.

Herein, we observed that serum iCa levels at both extremes were associated with increased short-term mortality, suggesting a U-shaped phenomenon between iCa and the short-term outcomes of acute ischemic stroke patients. However, iCa demonstrated a linear relationship with long-term ischemic stroke mortality, where only low serum iCa was related to poor long-term outcomes in acute ischemic stroke patients. Importantly, this study also observed that tCa was not related to outcomes in ischemic stroke patients, neither in terms of short-term nor long-term mortality.

The variations in results observed for serum tCa and iCa may find explanation in the physiological characteristics of Ca2+. In serum, Ca exists in 3 fractions: 50% in a biologically active ionized state, 40% conjugated to serum proteins (primarily albumin), and 10% bound to anions like citrate and bicarbonate [34]. Of these, only free calcium ions exhibit physiological activity [6]. Notably, as approximately 50% serum calcium is bound to proteins, alterations in protein concentration alone can cause changes in total Ca without impacting the physiologically and clinically significant ionized Ca. To address this, some studies resort to adjusting total serum Ca concentration for protein when directly measuring ionized Ca is not feasible [9, 11–15].

Several hypotheses have been proposed to elucidate the relationships between relatively low serum Ca levels and an elevated mortality rate. Firstly, low Ca levels may result from the influx of Ca into cells, a mechanism linked to ischemic cell death [15]. Lower calcium levels may indicate greater severity of AIS [35]. Secondly, diminished serum Ca levels can disrupt adhesion in endothelial cells, interrupting cell–cell adhesion, potentially compromising the integrity of the blood–brain barrier and leading to edema [36]. Thirdly, Ca ions are integral to coagulation factor IV, crucial in the entire coagulation process, low serum Ca levels may induce hemorrhagic transformation (HT) [37]. Fourth, serum Ca levels can contribute to the risk of stroke-associated infection when they fall below normal. Hypocalcemia may contribute to immune dysfunction, increasing the likelihood of infection [38]. Finally, the occurrence of low serum ionized calcium is linked to secondary hyperparathyroidism and inceased secretion of parathyroid hormone (PTH) [39]. Research has demonstrated that PTH is associated with various cardiovascular diseases, including endothelial dysfunction, vascular stiffness, calcification, and reduced elasticity of large arteries [40, 41].

Conversely, elevated iCa levels are associated with unfavorable long-term mortality following AIS, and several plausible explanations can be considered. Firstly, high Ca levels contribute to vascular calcification and atherosclerosis, indicative of a more adverse cerebrovascular foundation [42, 43]. Secondly, Ca ions serve as essential intracellular messengers and play a pivotal role in neuronal injury and cell death. Recent research has even unveiled the impact of Ca ions on cortical spreading depolarization after ischemic damage by modulating microglia activity [44]. However, it is noteworthy that, in this study, higher iCa levels did not exhibit prognostic significance for long-term mortality, and the underlying mechanism for this observation remains unknown.

This research has several advantages. Firstly, recognizing the limitations of tCa measurements in accurately identifying exact Ca derangements (given its dependence on serum albumin concentrations), the prognostic potential of both serum iCa and tCa was systematically analyzed. Secondly, the outcomes of univariate analysis were leveraged to select a comprehensive array of variables. Thirdly, the application of restricted cubic splines allowed for the exploration of potential non-linear relationships while accounting for confounding factors. Lastly, the study employed a crude model and three adjusted models incorporating various potential variables to scrutinize and ensure the stability of the results.

Nonetheless, this study comes with its own limitations. First, the assessment of serum iCa and tCa was conducted at a singular time point, making it challenging to ascertain the stability of serum calcium levels over time. Secondly, there is the potential for selection bias as some patients were excluded due to the absence of baseline serum iCa or tCa values and follow-up information. Despite our efforts to account for confounding factors through multivariate analysis, there may still exist unidentified variables that could impact the prognostic values of serum tCa and iCa. Furthermore, this study is of retrospective nature and performed in a singular academic medical center in the USA, which might limit the generalizability of our findings to other locations. Consequently, the validation of these results requires multicenter registry studies and prospective investigations.

## Conclusion

In conclusion, our findings suggest that serum ionized calcium, in contrast to total calcium, exhibits an association with IS prognosis. Notably, iCa demonstrated a U-shaped relationship with short-term IS mortality, where both low and high serum iCa levels were linked to poor short-term outcomes. However, iCa demonstrated a linear relationship with long-term IS mortality, with only low serum iCa associated with unfavorable long-term prognosis. To establish the robustness and clinical significance of these relationships, further studies, particularly large-scale prospective investigations, are imperative.

## Data Availability

Data in the article can be obtained from MIMIC-IV database (https://mimic.physionet.org/).

## Abbreviations

IS: Ischemic stroke
AIS: Acute Ischemic Stroke
iCa: Ionized Ca
tCa: Total Ca
MIMIC-IV: Medical Information Mart for Intensive Care
ICU: Intensive care unit
MAP: Mean artery pressure
SpO2: Percutaneous oxygen saturation
WBC: White blood cell count
eGFR: Estimated glomerular filtration rate
CAD: Coronary artery disease
CKD: Chronic kidney disease
SOFA: Sequential Organ Failure Assessment
APS III: Acute physiology score III
GCS: The Glasgow Coma Scale
SIRS: Systemic inflammatory response syndrome
RRT: Renal replacement treatment
ATP: Adenosine triphosphate
HT: Hemorrhagic transformation
PTH: Parathyroid hormone
